# Cyanoglobule lipid droplet accumulation as a stress response to nitrogen starvation in a non-N_2_-fixing mutant strain of *Anabaena sp. PCC 7120*

**DOI:** 10.1371/journal.pone.0343220

**Published:** 2026-02-20

**Authors:** Febri A. Susanto, Carrie Hiser, Mohit Mahey, Peter K. Lundquist

**Affiliations:** 1 Department of Biochemistry and Molecular Biology, Michigan State University, East Lansing, Michigan, United States of America; 2 Plant Resilience Institute, Michigan State University, East Lansing, Michigan, United States of America; 3 Department of Plant, Soil and Microbial Sciences, Michigan State University, East Lansing, Michigan, United States of America; Central University of Kerala, INDIA

## Abstract

Cyanoglobules are lipid droplets of cyanobacteria that share compositional and functional features with plastoglobules of plant chloroplasts. However, their roles in stress physiology remain poorly defined, particularly in filamentous cyanobacteria. Here, we characterize cyanoglobule dynamics and composition during nitrogen starvation in a non-diazotrophic derivative of *Anabaena* sp. PCC 7120. This experimental context enables analysis of sustained nitrogen deprivation in vegetative cells without the transient and heterogeneous effects of heterocyst differentiation and nitrogenase activity, while recognizing that the strain does not represent wild-type physiology. Nitrogen starvation induced striking morphological remodeling, including increased cyanoglobule size and abundance. Proteomic analysis revealed a cyanoglobule proteome enriched in homologs of the plant plastoglobule proteome as well as other redox regulators and isoprenoid metabolism enzymes, pointing to roles in pigment turnover and stress adaptation. Lipidome profiling revealed high levels of plastoquinone derivatives and other prenyl-lipid species. Collectively, our findings establish cyanoglobules as dynamic and stress-responsive compartments associated with redox and lipid remodeling during nutrient limitation. By leveraging a non-diazotrophic and comparative analysis, we show that key features of cyanoglobule formation and composition are observed independently of heterocyst differentiation and parallel those described in other cyanobacterial species and plant plastoglobules.

## Introduction

Plastoglobule lipid droplets of plants, once regarded as passive lipid storage structures, are now recognized as dynamic organelles involved in redox regulation, metabolic homeostasis, and cellular stress responses [1–5]. In filamentous cyanobacteria such as *Anabaena* sp., lipid droplets have primarily been studied in the context of nitrogen fixation, with particular attention to their presence in heterocysts. These specialized cells facilitate atmospheric nitrogen assimilation and undergo extensive metabolic and structural changes during nitrogen starvation [6]. As a result, the regulatory mechanisms underlying lipid droplet formation in *Anabaena* have traditionally been interpreted within the framework of diazotrophic metabolism.

Recent advances have expanded this perspective. In *Synechocystis*, a unicellular cyanobacterium lacking nitrogen fixation capability, a distinct population of lipid droplets known as cyanoglobules has been identified. These structures exhibit proteomic and lipidomic profiles similar to plastoglobules in plant chloroplasts, suggesting an evolutionary link and shared functional properties [7]. The presence of cyanoglobules in non-diazotrophic cyanobacteria supports the hypothesis that such droplets may serve roles beyond carbon storage, potentially contributing to stress adaptation and regulation of photosynthetic activity.

A foundational study by Peramuna and Summers [8] first demonstrated the presence of lipid droplets throughout vegetative cells in the filamentous cyanobacterium *Nostoc punctiforme*, highlighting their enrichment in triacylglycerols, carotenoids, and alkanes. This work provided the first detailed characterization of cyanobacterial lipid droplets and emphasized their potential physiological relevance outside the specialized context of nitrogen fixation. However, the extent to which these structures resemble cyanoglobules or plastoglobules remained unclear, particularly in filamentous backgrounds that lack nitrogen fixation capacity.

Nitrogen deprivation is a well-established trigger for metabolic reprogramming in cyanobacteria. In non-nitrogen-fixing strains such as *Synechocystis* and *Synechococcus*, nitrogen limitation induces lipid accumulation independent of heterocyst formation. This includes the formation of polyhydroxybutyrate (PHB) granules as well as other inclusions such as cyanophycin granules [9–11]. These findings suggest that lipid droplet formation may be a conserved cellular response to nitrogen stress across cyanobacterial lineages. However, the dynamics and molecular composition of cyanoglobules in filamentous cyanobacteria that lack nitrogen fixation remain unexplored. In *Anabaena* sp., research on lipid droplets has remained focused on diazotrophic conditions, leaving a gap in understanding how these structures behave in non-nitrogen-fixing strains.

This study investigates whether cyanoglobules are present in *Anabaena* sp. PCC 7120 under nitrogen deprivation in the absence of nitrogen fixation. Using a non-nitrogen-fixing strain, we examine cyanoglobule accumulation and characterize their molecular composition. The study also considers whether cyanoglobules in *Anabaena* share similar features with those observed in *Synechocystis*, or if they represent a distinct lipid droplet population. By removing the confounding influences of heterocyst differentiation and nitrogenase activity, this experimental system provides a context to investigate cyanoglobule dynamics as a direct response to nitrogen stress. The findings presented here offer new insight into the regulation and function of cyanoglobules in a filamentous background and contribute to a broader understanding of lipid droplet biology in cyanobacteria. Furthermore, this work establishes a descriptive foundation for future studies, including synthetic biology application, where defined non-diazotrophic strains are often favored.

## Materials and methods

### Strain, growth condition, and treatment

An *Anabaena* strain that lost its nitrogen fixation ability (*Anabaena*^ΔN^ strain) was generated by culturing cells through ten successive sub-culturing steps in nitrogen-rich medium and subsequently testing growth under nitrogen-deficient conditions. Loss of diazotrophic capacity was confirmed by growth arrest under nitrogen-depleted conditions.

For staining and visualization of cyanoglobules, starter cultures were incubated at 32 °C with continuous illumination (150 μmol photons m^–2^ s^–1^) using Sylvania 15W Grolux cool white bulbs, under shaking at 150 rpm with 2% CO₂ supplementation, inside a Multitron Incubator (Infors HT, Bottmingen, Switzerland). Cultures were grown for two days and harvested at either logarithmic (3–5 µg chl a mL^–1^) or stationary phase (8–50 µg chl a mL^–1^).

For N-deficiency treatment, triplicate starter cultures at logarithmic phase (OD_750_ 1.2) were centrifuged at 4700 rpm for 3 minutes and the resulting cell pellets were resuspended in nitrogen-limited BG11 medium (0.55 mM N; BG11^–N^). For control conditions, resuspension was made in standard BG-11 medium containing 17.6 mM NaNO_3_. Modified BG11^–N^ was prepared by reducing NaNO_3_ and replacing the equivalent sodium using NaCl to maintain ionic balance (final Na⁺ concentration = 17.05 mM). Cultures were maintained until they reached stationary phase (ca. 7 days) before sample collection.

### Genome isolation, sequencing, and analysis

Genomic DNA from wild-type (Anabaena^WT^) and mutant *(Anabaena*^ΔN^) strains was sequenced using Oxford Nanopore long-read sequencing (Plasmidsaurus Inc., South San Francisco, USA). Raw reads were filtered with Filtlong v0.3.1 (Wick, 2018), retaining only reads ≥1,000 bp in length and the top 95% of reads by quality to remove short or low-quality reads that may bias downstream analyses. The filtered reads were aligned to the *Anabaena* sp. PCC 7120 reference genome [12] using Minimap2 v2.26-r1175 [13] with the “-ax map-ont” preset, which is optimized for long-read sequencing. Alignment files in SAM format were converted to BAM, sorted and indexed using SAMtools v1.19.2 [14]. Variants were then called with Clair3 [15], a deep-learning–based variant caller that has been benchmarked to outperform traditional pipelines on nanopore data [15], using default parameters with the "--include_all_ctgs" flag to enable variant detection across all contigs and the "--no_phasing_for_fa" flag to correctly model the haploid genome of *Anabaena*. Variant call files (VCFs) were filtered to retain only high-confidence variants with QUAL ≥10, followed by normalization with BCFtools v1.19 [14] to standardize multi-allelic and indel representations across samples. Variants unique to the mutant strains were extracted by removing those shared with wild-type, ensuring the analysis focused specifically on mutant-associated mutations. Functional annotation was performed with SnpEff v5.2c [16] to predict coding and protein-level impacts, and SnpSift v5.2c [16] was used to filter and manipulate annotations for prioritizing variants of interest. All commands and scripts used in this analysis are available on GitHub at https://github.com/mohitmahey/Anabaena_variant_calling_ONT_reads.

### Stress treatments

Starter cultures of *Anabaena*^ΔN^ were grown in 125-mL flasks of BG11 media at 25 °C with shaking at 150 rpm under 13–15 µmol photons s^−1^ m^−2^ from cool white, fluorescent lights for 2 days. For nutrient stresses, starter culture was harvested, pelleted and aliquots resuspended in BG11^-N^ (0.55mM; for N stress), low P media BG11^-P^ (0.0072mM; for P stress), or in standard BG11 media (for control) and grown until stationary phase (8–50 µg Chl a mL^−1^), at which point samples were collected for CLSM and TEM. For dark stress, starter culture was harvested, pelleted and aliquots resuspended in BG11 medium and wrapped completely in aluminum foil and grown for an additional 7 days, at which point samples were collected for CLSM and TEM. Independent triplicate cultures were sampled and imaged by CLSM and TEM.

### Microscopy of cell morphology

*Anabaena*^WT^ and *Anabaena*^ΔN^ strains were cultured under two conditions: nitrogen-free BG-11^-N^ medium and nitrogen-replete BG-11 medium. For microscopy, 1 mL of culture was harvested by centrifugation at 3,000 × g for 5 min, and the pellet was gently resuspended. A 5 μL aliquot of the cell suspension was placed onto a clean glass microscope slide. To immobilize the filaments and maintain hydration during imaging, a thin agarose pad (1.5% [w/v] agarose in BG-11 medium, cut into ~0.5 cm squares) was placed carefully on top of the cell suspension.

Samples were imaged using a Thunder Imager System (Leica Microsystems, USA) equipped with a fluorescence light source and a high-sensitivity sCMOS camera. Images were acquired with a 100 × oil-immersion objective using phase contrast to visualize vegetative cells and heterocysts. Acquisition parameters were kept constant across *Anabaena*^WT^ and *Anabaena*^ΔN^ strains to allow direct comparison. Images were processed with the Leica Thunder image software to reduce background and improve contrast.

### Confocal laser scanning microscopy (CLSM) of cyanoglobule lipid droplets

To visualize intracellular cyanoglobules lipid droplets, cyanobacterial pellets from 2 mL of culture were stained with the lipophilic dye monodansyl pentane (MDH). MDH was diluted to a final concentration of 0.1 mM from a 1 mM DMSO stock, and cells were incubated in this solution for 1–2 minutes. Stained cells were immobilized on 1% agarose gel pads and mounted on glass slides for imaging. Fluorescence signals were acquired using an Olympus FLUOVIEW FV1000 confocal microscope with a 100x UPlanApo oil objective (NA 1.40). MDH was excited at 444 nm and emissions were captured at 485–535 nm. Chlorophyll autofluorescence was visualized by excitation at 647 nm and collection from 662–737 nm. Time-series images were collected every 10 seconds over a 30-minute interval.

### Transmission electron microscopy (TEM)

For ultrastructural analysis, 1 mL of culture was pelleted at 14,000 × g for 1 minute and embedded in 8% agarose. Embedded samples were fixed with 2% glutaraldehyde and 2% paraformaldehyde in 0.05 M cacodylate buffer (pH 7.3), followed by three 10-minute washes. Post-fixation was performed with 1% osmium tetroxide for 2 hours. Dehydration was carried out through graded acetone steps (30% to 100%, each for 15 minutes) and repeated with final dehydration in 100% acetone three times.

Samples were embedded sequentially in Spurr’s resin, progressing from 20%, 50%, 75%, and finally 100% resin, with overnight incubation at each step. After three additional changes in fresh 100% resin (one per day), samples were polymerized at 60 °C for 24 hours. Ultrathin sections (60–90 nm) were prepared using a Power Tome XL ultramicrotome (RMC Boeckeler, Tucson, USA), mounted on Formvar-coated 150 mesh copper grids, and stained with 2% uranyl acetate followed by Reynolds’s lead citrate. Imaging was carried out using a JEOL JEM-1400 Flash transmission electron microscope equipped with a Metataki Flash CMOS camera. Measurements of cyanoglobule size and number were quantified using ImageJ.

### Isolation of cyanoglobules

Isolation of cyanoglobule fractions was performed based on a previously published protocol [17] with slight modifications. Stationary phase cultures (50 mL, OD750 ~ 2.0) were pelleted and washed twice in Buffer A (25 mM HEPES-KOH pH 7.8, 250 mM sucrose). Cell disruption was achieved by three successive passes through a French Press (AMINCO FA-028) at 1100 psi. Cell lysates were centrifuged at 150,000 × g for 30 minutes at 4 °C after overlaying the homogenate with medium R (50 mM HEPES-KOH pH 8.0, 5 mM MgCl₂). The resulting floating lipid-rich layer was collected as crude cyanoglobules.

To further purify cyanoglobules, the crude fraction was mixed with medium R containing 0.7 M sucrose and layered beneath medium R containing 0.2 M sucrose. This gradient was centrifuged at 150,000 × g for 90 minutes at 4 °C. The resulting cyanoglobule fraction was harvested, lyophilized, and stored at –80 °C. All buffers contained protease and phosphatase inhibitor cocktails (74 μm antipain, 130 μm bestatin, 16.5 μm chymostatin, 56 μm E64, 2.3 μm leupeptin, 37 μm phosphoramidon, 209 μm AEBSF, 0.5 μm aprotinin, 50 mm NaF, 25 mm β-glycerophosphate, 1 mm Na-orthovanadate and 10 mmNa-pyrophosphate) to preserve protein integrity.

### Proteomics analysis of purified cyanoglobules from the *Anabaena*^ΔN^ strain

Lyophilized cyanoglobule samples were reconstituted in SDS-PAGE buffer, heated at 60 °C for 10 minutes, and loaded on 12.5% BioRad Criterion gels. After brief electrophoresis (50 V for ~20 min), proteins were visualized using the Pierce Silver Stain kit (Thermo Fisher, Waltham, USA). Proteins were excised for in-gel digestion.

Protein digestion followed a modified Shevchenko protocol [18]. Bands were dehydrated in acetonitrile, reduced with DTT (10 mM in 100 mM ammonium bicarbonate) at 56 °C for 45 min, alkylated with 50 mM iodoacetamide, and rehydrated in trypsin (0.005 µg/µL) overnight at 37 °C. Peptides were extracted using 60% acetonitrile with 1% TFA and dried via vacuum centrifugation.

Peptides were then resuspended in 100 mM TEAB and labeled using Thermo Scientific 11-plex TMT reagents per the manufacturer’s instructions. Labeled peptides were cleaned using C18 SepPak cartridges, dried, and resuspended in 2% acetonitrile with 0.1% TFA. Peptides were separated on a Thermo EASY-nLC 1200 system using a PepMap RSLC trap and resolving column, with a 45-minute gradient and 300 nL/min flow rate.

Mass spectrometry was performed using a Q Exactive HF-X mass spectrometer in data-dependent acquisition mode. MS1 resolution was 120,000 (m/z 200), followed by HCD fragmentation and MS2 acquisition at 45,000 resolution. Data were processed with MaxQuant v1.6.15.0 [19], using as isobaric labels the ‘TMT10plex-Lys130C’, TMT10plexL131N’ and ‘TMT11plex-Lys131C’. Methionine oxidation, Ser/Thr/Tyr phosphorylation and N-terminal acetylation were set as variable modifications. Carbamidomethylation was set as fixed modification. Data was searched against a custom-built *Anabaena* protein database that concatenates protein sequence predictions from our genomic sequencing of the *Anabaena*^ΔN^ strain and the *Anabaena*^WT^ strain (12287 protein entries) with a reversed-sequence decoy database constructed bin the MaxQuant pipeline to estimate false discovery rate. Peptide and protein-level false-discovery rate was set to 1%. The mass spectrometry proteomics data have been deposited to the ProteomeXchange Consortium via the PRIDE partner repository [20] with the dataset identifier PXD069172.

### Lipidomics analysis by LC-MS/MS

Immediately following lyophilization, lipid extraction was carried out using 300 µL of a solvent mixture consisting of methanol, chloroform, and formic acid in a 20:10:1 ratio (v/v/v). To induce phase separation, 150 µL of a buffer containing 0.2 M phosphoric acid and 1 M potassium chloride was added, and samples were centrifuged at 13,000 × g for 1 minute at room temperature. The organic (chloroform) layer, containing the extracted lipids, was carefully collected, evaporated under a nitrogen gas stream, and reconstituted in 100 µL of high-purity isopropanol. The resulting lipid extracts were filtered with 0.45-µm PTFE syringe filter (Restek Corp., Bellefonte, USA) and transferred into amber HPLC vials equipped with glass inserts to prevent adsorption loss.

Lipidomic profiling was performed using a Thermo Scientific Q-Exactive hybrid quadrupole-Orbitrap mass spectrometer integrated with a Vanquish ultra-high-performance liquid chromatography (UHPLC) system operating in positive ion electrospray ionization mode. Chromatographic separation was achieved on a C18 reverse-phase Acquity column (Waters) over a 20-minute run. The mobile phases included Solvent A (acetonitrile:water 60:40, v/v) and Solvent B (isopropanol:acetonitrile 90:10, v/v), both containing 0.1% formic acid and 10 mM ammonium formate. The elution program consisted of the following gradient: 20% B for the first 2 minutes, ramping to 43% B from 2.1 to 11.9 minutes, 54% B at 12 minutes, followed by 70% B from 12.1 to 18 minutes, then 99% B at 18.1 to 19 minutes, and re-equilibration to 20% B at 20 minutes. The injection volume was 10 µL, with a flow rate of 0.4 mL/min, and the column temperature maintained at 55 °C.

MS1 full scan spectra were acquired in the m/z 200–2000 range, and data-dependent MS2 fragmentation was performed for the five most intense precursor ions per scan cycle. Data acquisition and peak detection were optimized for retention time precision and mass accuracy.

Acquired data files were processed using Progenesis QI software version 2.3 (Nonlinear Dynamics, Newcastle-upon-Tyne, UK). The software was used to perform baseline correction, retention time alignment, peak detection, and normalization. Statistical filtering of the identified features was carried out with a threshold of p < 0.01 and q < 0.01 to retain high-confidence lipid species. Lipid annotation was performed by matching accurate mass and fragmentation spectra against the LipidBlast library within Progenesis QI and further validated by comparison with reference spectra from LipidMaps and the Metabolomics Workbench.

To distinguish lipid profile changes between treatment groups, multivariate statistical analysis was conducted using orthogonal partial least squares discriminant analysis (OPLS-DA) in EZinfo version 3.0.3 (Sartorius, Göttingen, Germany). Lipid enrichment and class-level distributions were calculated based on normalized ion intensities and visualized graphically for comparative interpretation.

## Results

### Development of a non-N2-fixing strain of *Anabaena*

An *Anabaena* strain that lost its nitrogen fixation ability (*Anabaena*^ΔN^) was identified following successive sub-culturing of cells at least ten times through nitrogen-replete BG11 medium and subsequently testing growth under nitrogen-deficient conditions (BG11^-N^). Genome sequencing and SNP analysis revealed mutations in several genes important for heterocyst formation and function (S1 Table). Among them, there were three genes essential for growth in the absence of nitrogen: *alr2817 (hetC), alr2887 (devB), and alr0952 (coxC)*. These genes are required for differentiation of heterocysts or protection of the nitrogenase from oxidation [21–24].

In addition to these three core genes, four other candidates—*alr3956 (ndhF), alr3355 (ndhH), all4019 (zwf), and alr0693*—may also contribute to the inability of *Anabaena*^ΔN^ strain to fix nitrogen and potentially influence other aspects of cellular physiology. These genes are involved in supporting heterocyst metabolism. A summary of the whole genome sequence results of *Anabaena*^WT^ and *Anabaena*^ΔN^ is presented in S1 Fig.

### Increased size and abundance of *Anabaena*^ΔN^ cyanoglobules under various stresses

**Fig 1:**
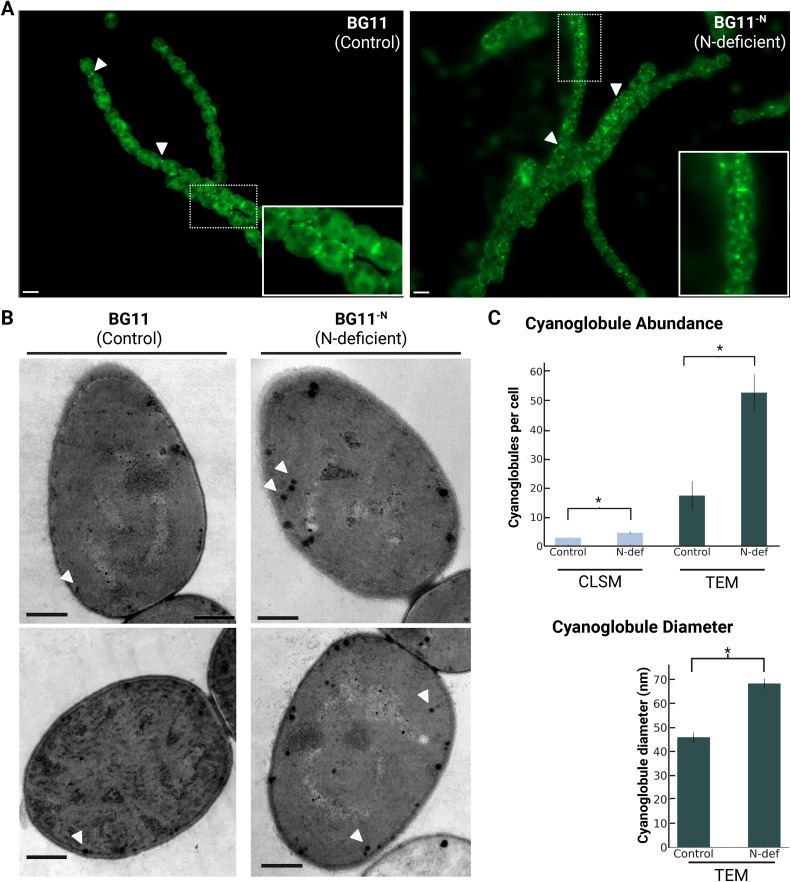
Visualization and quantification of cyanoglobules in *Anabaena*^ΔN^ cells under nitrogen deficiency. **(A)** Representative confocal laser scanning microscopy (CLSM) images showing cyanoglobule accumulation stained with monodansyl pentane (representative cyanoglobules are indicated with white arrowheads) in control (17.6 mM N; BG11,) and nitrogen-deficient conditions (0.55 mM N; BG11^-N^). Scale bars indicate 2 µm. **(B)** Transmission electron micrographs (TEM) illustrating osmiophilic cyanoglobule structures (indicated by white arrowheads) in *Anabaena*^WT^ and *Anabaena*^ΔN^. Scale bars indicate 500 nm. **(C)** Quantitative analysis of cyanoglobule abundance (number of cyanoglobules per cell) obtained from CLSM and TEM data, and diameter measurements (nm) derived from TEM. Data represent the mean ± 1 standard deviation of three biological replicates (*n* = 3). Statistical significance was determined using a two-tailed unpaired Student’s *t*-test; asterisks indicate statistically significant differences of p < 0.05.

We first tested cyanoglobule proliferation under a suite of three different stressors, N-deficiency, P-deficiency and darkness (**Fig 1**, S2 Fig). To examine cyanoglobule accumulation, *Anabaena*^ΔN^ cells were stained with monodansyl pentane (MDH), commonly used for lipid droplet detection [25].

CLSM observations demonstrated that all three stresses promote greater numbers of cyanoglobules per cell, particularly for P-deficiency and N-deficiency (**Fig 1A**, S2 Fig, S2 Table). Transmission electron microscopy (TEM) confirmed this trend, revealing darkly stained osmiophilic lipid globules consistent with the previously described cyanoglobule morphology in *Synechocystis* [7] (**Fig 1B & 1C**, S2 Fig, S2 Table). Notably, the average diameter of cyanoglobules also increased substantially under each of the stresses. Again, this was most striking for P-deficiency and N-deficiency (**Fig 1B 1C**, S2 Fig, S2 Table). Thus, both microscopic methods revealed consistent trends in cyanoglobule abundance and size, reinforcing the conclusion that cyanoglobule formation and accumulation are markedly enhanced under stress.

### Growth and pigmentation phenotypes of *Anabaena*^ΔN^

**Fig 2:**
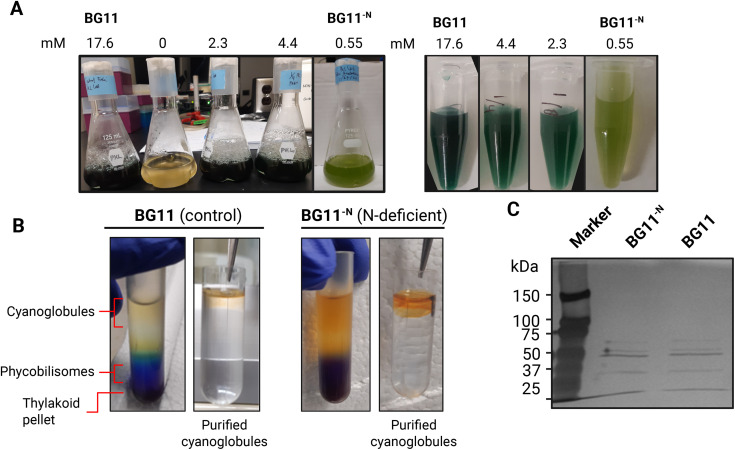
Isolation and purification of cyanoglobules from *Anabaena*^ΔN^ grown in control and N-deficient media. **(A)** Growth and pigmentation phenotypes of *Anabaena*^ΔN^ under control (BG11) and varying levels of N-deficiency. **(B)** Separated cell lysate (left panel, sucrose gradient) and floated cyanoglobules (right panel, sucrose gradient) from cultures grown in BG11 and BG11^-N^ media. Note that yellowish lipid pads were obtained from control BG11 cultures, while bright-orange pads were isolated from nitrogen-deficient BG11^-N^ conditions. The final purified cyanoglobule fraction appeared as a single lipid pad atop the sucrose gradient. **(C)** Silver staining of SDS-PAGE demonstrates a common banding pattern of isolated cyanoglobules from cells grown under both control and N-deficient conditions.

Because nitrogen availability strongly influences photosynthetic metabolism and lipid storage, we used nitrogen deprivation as a physiological model to investigate how cyanoglobules respond to nutrient stress. In *Arabidopsis*, nitrogen limitation leads to an increase in both the size and number of plastoglobules, reflecting enhanced lipid remodeling [26]. Thus, we continued our investigation by focusing on the composition and behavior of cyanoglobules under N-deficiency. After transferring *Anabaena*^ΔN^ into nitrogen-deficient media (0.55 mM nitrogen; BG11), compared to control conditions (17.6 mM nitrogen; BG11^-N^), distinct physiological changes were evident. By day four of nitrogen deprivation, cells exhibited lighter pigmentation compared to control growth conditions (**Fig 2A**), indicative of chlorosis. This visual phenotype aligned well with optical density (OD_750_) and chlorophyll *a* levels (S3 Fig), and were consistent with the previous reports of other cyanobacterial species under nutrient deficiency [27–29].

To examine whether *Anabaena*^ΔN^ exhibits altered cell morphology, we compared *Anabaena*^WT^ and *Anabaena*^ΔN^ under nitrogen-depleted conditions. In the wild-type, growth in BG11^-N^ medium without nitrogen supplementation led to the expected formation of heterocysts, which were clearly distinguishable along the filaments (S4A Fig). In contrast, the ΔN strain displayed heterogeneous morphologies, including filaments with normal-appearing cells as well as abnormally enlarged and elongated cells (S4B Fig). Additionally, while wild-type cultures remained as a uniform green suspension, the ΔN strain consistently developed clumps in liquid culture, indicating an altered physiological response to nitrogen starvation. Although we cannot definitively determine whether the observed morphological changes arise directly from the loss of the targeted genes or represent a secondary response to the inability to fix atmospheric N₂, the results highlight that *Anabaena*^ΔN^ has a clear defect in N metabolism that manifests in both filament organization and culture-level behavior.

The increased size and number of cyanoglobules observed in *Anabaena*^ΔN^ cells subjected to nitrogen deficiency align closely with previous observations in *Synechocystis* under phosphorus deprivation, and parallel the stress-induced plastoglobule formation commonly reported in land plants [30]. These findings collectively suggest a conserved and dynamic role of cyanoglobules as lipid reservoirs or stress-responsive organelles, modulating cellular responses under nutrient-limited conditions.

### Isolation and purification of cyanoglobules from *Anabaena*^ΔN^

To examine the biochemical composition of cyanoglobules under nitrogen-limiting conditions, cyanoglobules were isolated from *Anabaena*^ΔN^. Cultures (50 mL) were grown under nitrogen-deficient BG11^-N^ medium to impose a N-deficient stress until they reached an optical density (OD₇₅₀) of 2.0 per mL, the optimal stage for harvesting cyanoglobule-rich cells (S3 Fig).

Cells subjected to nitrogen deficiency displayed visibly lighter pigmentation (**Fig 2A**), indicative of chlorosis, and had reduced chlorophyll *a* content (2.6 ± 0.4 µg chl *a* mL^-1^) compared to control cultures (3.5 ± 0.6 µg chl *a* mL^-1^). These differences occurred despite the cultures having comparable cell densities based on optical density measurements. This visual and biochemical shift in pigmentation provided an indication of physiological stress and cyanoglobule enrichment.

Following cell harvest, a crude cyanoglobule fraction was obtained via two-step differential centrifugation. As illustrated in **Fig 2B**, the initial step produced two distinct lipidic layers, visible as yellowish (control) or bright orange (nitrogen deficient) zones, suggesting compositional differences in pigment or lipid content. The two lipid pads from each growth condition were pooled and subjected to further purification by sucrose gradient centrifugation. This process resulted in a single, dense lipid pad floating at the top of the gradient, corresponding to enriched cyanoglobules.

To validate the co-isolation of proteins with the isolated cyanoglobule fraction, samples were resolved using SDS-PAGE and visualized by silver staining. As shown in **Fig 2C**, multiple protein bands were observed in both control and N-deficient cultures. The purified cyanoglobules were subsequently subjected to proteomic and lipidomic analyses for in-depth characterization of the molecular composition.

### Proteomic profiling of cyanoglobules in *Anabaena*^ΔN^

To investigate the proteomic composition of cyanoglobules in *Anabaena*^ΔN^, we conducted TMT-based quantitative proteomic analysis of purified cyanoglobules isolated from cells grown under both control and nitrogen-deficient conditions. A total of 97 proteins were confidently annotated and quantified at a peptide and protein level false discovery rate of 1% (S3 Table). Thirteen proteins identified in the isolated cyanoglobules have orthologs in the *Synechocystis* sp. PCC 6803 cyanoglobule proteome [7] and/or the *A. thaliana* plastoglobule proteome [5], pointing to the commonality in the composition across disparate cyanobacterial species [7,31–33]. We considered these thirteen proteins to represent the putative cyanoglobule proteome of *Anabaena*.

The most abundant and differentially enriched proteins under N-deficiency were homologs of NAD(P)H dehydrogenase C1 (NDC1, alr4094) and Plastoglobular protein 18 (PG18, alr4514), with log_2_ fold-changes of 4.28 and 4.80, respectively (**Table 1**). These two proteins are well-established plastoglobule components in *A. thaliana* [5,33] and *Synechocystis* [7]. Interestingly, ndbB is also the most abundant protein of the cyanoglobule core proteome in *Synechocystis* sp. PCC 6803 [4]. NDC1 is essential for electron transport and plastoquinone redox homeostasis. *A. thaliana* ndc1 mutants exhibit elevated oxidative stress, altered plastid lipids and early bolting, reflecting its importance in plastidic metabolic regulation [34–36]. Similarly, functional studies have shown that PG18 mutants display thylakoid disorganization, reduced photosystem efficiency, and increased photodamage due to impaired chloroplast structure and photoprotective capacity [37].

**Table 1:** Dynamic changes to the *Anabaena*^ΔN^ cyanoglobule proteome under N-deficiency.

*Anabaena 7120* Gene ID	*Synechocystis 6803* Gene ID	*A. thaliana* Gene ID	Fraction of proteome (%)		Fold-change (Log_2_)	Protein Annotation
			Control	N-def		
*alr4094/ndbB*	*slr1743/ndbB*	*At5g08660*	0.76	14.83	4.28	NDC1; NAD(P)H dehydrogenase C1
*all5305*	*RS0201695*	*At4g35250*	1.13	14.17	3.65	SDR family oxidoreductase
*alr4514*	*sll1769*	*At4g13200*	0.49	13.66	4.8	PG18; Plastoglobular protein of 18kD
*alr1709*	*sll0553*	*At5g19850*	0.76	9.55	3.64	alpha/beta hydrolase
*alr1788*	*NA*	*At1g32220*	0.31	5.36	4.12	FAD-dependent oxidoreductase
*asl4034*	*ssl0294*	*At1g28150*	0.23	5.11	4.47	hypothetical protein
*all0736*	*NA*	*At1g28100*	0.27	4.46	4.06	acetoacetate decarboxylase family protein
*all0772*	*NA*	*At2g34460*	0.24	3.66	3.93	class I SAM-dependent methyltransferase
*alr2751*	*sll1218*	*At2g34460*	0.27	1.83	2.74	SDR family oxidoreductase
*alr4318*	*sll1568*	*At4g22240*	0.13	1.74	3.7	FBNβ; PAP/fibrillin family protein
*all1225*	*slr0545*	*At1g06690*	0.03	0.73	4.73	aldo/keto reductase-like
*all3744/crtO*	*slr0088/crtO*	*At5g49555*	0.02	0.21	3.73	β-carotene 4-monoketolase
*all4960/spkH*	*sll0005/spkH*	*At1g79600*	0.03	0.19	2.43	ABC1K3; protein serine/threonine kinase

Proteins identified as plastoglobule core protein homologs of Arabidopsis are marked in italic font. Annotations were curated by the authors based on sequence homology and nomenclature from databases including the Kyoto Encyclopedia of Genes and Genomes (KEGG), UniProt, and the Plant Proteome Database. Proteins are sorted by their abundance under nitrogen-deficient (N-def) conditions.

Alongside these core proteins, other conserved plastoglobule markers were also enriched in cyanoglobules. The identification of Fibrillin 2 (FBNβ, all4697) and ABC1K3 (all4960) supports the conserved nature of these structures [33]. While two FBN isoforms are encoded in *Anabaena*, only FBNβ was detected in the cyanoglobules, paralleling findings from *Synechocystis* where only one FBN isoform (FBNβ) is cyanoglobule-localized. Likewise, ABC1K3 was the sole member of the four encoded ABC1 kinases present in the cyanoglobules, suggesting selective localization and a conserved role in plastoglobule-related signaling or lipid metabolism.

In addition to these canonical components, several proteins involved in redox regulation and stress response were also identified. Notably, an aldo/keto reductase-like protein (AKR-like, all1225) and multiple short-chain dehydrogenase/reductase (SDR) family members such as all5305 and alr2751 exhibited high enrichment, with log₂ fold-changes of 3.65 and 2.74, respectively. These findings support the role of cyanoglobules in managing oxidative stress, a well-characterized function of plastoglobules [2,34]. Additional NAD(P)-binding oxidoreductases, including flavin reductase-related 1 (alr1788) and flavin reductase-related 2 (all0772/alr2751), were also abundant, indicating active redox metabolism within the compartment.

Interestingly, several proteins associated with pigment and lipid metabolism were highly enriched under nitrogen deficiency. The Neoxanthin-deficient 1 (NXD1, all0736) homolog showed a strong increase under N-deficiency (log₂ FC ~ 4.06), suggesting a role for cyanoglobules in carotenoid biosynthesis or regulation during nitrogen stress. Similarly, α/β-hydrolase (alr1709) and β-carotene ketolase (all3744) were enriched (log₂ FC 3.64 and 3.73, respectively), further implicating cyanoglobules in pigment remodeling and lipid turnover processes.

The dataset also included proteins with less-defined or potentially novel functions. Among them, UPF0426 protein (asl4034) was highly enriched (log₂ FC ≈ 4.8). Although UPF0426 is not typically associated with plastoglobules in higher plants, its possible localization to cyanoglobules in *Anabaena* suggests a specialized adaptation in filamentous cyanobacteria. Together, these observations highlight a conserved yet functionally diversified proteome architecture, where core lipid-metabolic and redox proteins are supplemented by lineage-specific factors to accommodate the physiological needs of *Anabaena* [2,28,30,36,38].

Taken together, these results reveal a cyanoglobule proteome in *Anabaena* that shares several conserved features with land plant plastoglobules, including lipid metabolism, redox regulation, and stress response functions. At the same time, the presence of unique or previously unreported proteins suggests that cyanoglobules have evolved species-specific adaptations, potentially reflecting differences in environmental responses and plastidial function in cyanobacteria compared to the modern plants.

### Lipidomic profiling of cyanoglobules in *Anabaena*^ΔN^

**Fig 3:**
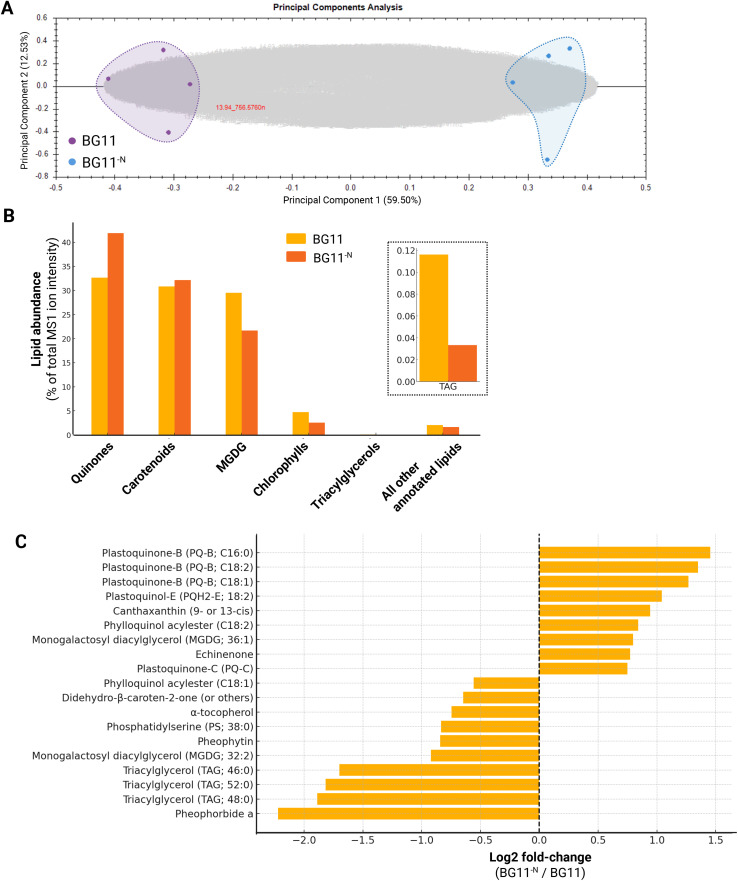
Lipidomic analysis of cyanoglobules from *Anabaena*^ΔN^ under nitrogen-deficient conditions. **(A)** Principal Components Analysis of cyanoglobule lipidomes under control (BG11) and nitrogen-deficient (BG11^-N^) conditions. Each colored dot represents a biological replicate (n = 4), each gray dot represents an individual lipid species. The clear separation along Principal Component 1 reflects the highly distinct lipid profiles between growth media. **(B)** Total lipid class abundance in cyanoglobules of *Anabaena*^ΔN^ measured as % of total MS1 ion intensity of all annotated lipids. Inset: TAG content shown on a separate axis due to lower relative abundance. **(C)** Fold-changes of specific lipid species in cyanoglobules of *Anabaena*^ΔN^ grown in BG11^-N^ relative to BG11 media. Selected lipids that change with statistical significance are presented (p < 0.05, Student’s t-test, n = 4 biological replicates, each of which is an independently grown culture). Several plastoquinone (PQ) and plastoquinol (PQH_2_) species were significantly upregulated, particularly PQ-B (C16:0, C18:1, C18:2) and PQH_2_-E (18:2). In contrast, chlorophyll breakdown products (pheophorbide a, pheophytin), MGDG species, TAGs, and α-tocopherol were markedly reduced.

Plastoglobules in land plants and their cyanobacterial analogs, cyanoglobules, are known to host specialized lipid classes such as prenyl-lipids, redox-active quinones, and stress-associated carotenoids [3–5,7,38]. To investigate how nitrogen deprivation affects the cyanoglobule lipidome in *Anabaena*^ΔN^, we performed LC-MS/MS-based lipidomic analysis on cyanoglobules isolated from cells grown under control and nitrogen-deficient conditions. The results reveal that nitrogen stress triggers extensive lipid remodeling within cyanoglobules, marked by the accumulation of plastoquinone derivatives and specific carotenoids, and a concurrent depletion of thylakoid-associated lipids such as MGDG, TAGs, and chlorophyll breakdown products (**Fig 3**, **Table 2**, S4 Table).

**Table 2:** LC-MS-based quantification of cyanoglobule lipids under N-deficiency.

Lipid Class	Lipid Species ^a^	Normalized abundance (%)		Fold-change (Log_2_) ^b^
		Control	N-def	
Quinones	Menaquinone-6	0.001	0.0018	***0.76***
Quinones	Phylloquinol acylester (C18:1)	0.1731	0.2738	***0.66***
Quinones	Phylloquinol acylester (C18:1)	0.0026	0.0018	***−0.59***
Quinones	Phylloquinol acylester (C18:2)	0.1026	0.1831	***0.84***
Quinones	Plastochromanol-8 (PCH2–8)	0.0018	0.002	0.16
Quinones	Plastoquinol-B (PQH2-B; C16:0)	1.5082	1.758	0.22
Quinones	Plastoquinol-B (PQH2-B; C18:0)	0.636	0.7448	0.23
Quinones	Plastoquinol-C (PQH2-C)	0.0846	0.1011	0.26
Quinones	Plastoquinol-E (PQ-E; 18:0)	5.7214	7.3356	0.36
Quinones	Plastoquinol-E (PQH2-E; 16:0)	14.1795	15.2657	0.11
Quinones	Plastoquinol-E (PQH2-E; 18:1)	3.7231	5.4967	***0.56***
Quinones	Plastoquinol-E (PQH2-E; 18:2)	1.8118	3.7185	***1.04***
Quinones	Plastoquinone-A (PQ-9)	3.3271	4.7307	***0.51***
Quinones	Plastoquinone-A (PQ-9)	0.345	0.3293	−0.07
Quinones	Plastoquinone-B (PQ-B; C16:0)	0.1011	0.2764	***1.45***
Quinones	Plastoquinone-B (PQ-B; C18:0)	0.5304	0.7119	0.42
Quinones	Plastoquinone-B (PQ-B; C18:1)	0.0376	0.0907	***1.27***
Quinones	Plastoquinone-B (PQ-B; C18:1)	0.2931	0.6853	***1.23***
Quinones	Plastoquinone-B (PQ-B; C18:2)	0.0026	0.0068	***1.39***
Quinones	Plastoquinone-B (PQ-B; C18:3)	0.0431	0.0547	0.34
Quinones	Plastoquinone-C (PQ-C)	0.0445	0.0747	***0.75***
Quinones	Plastoquinone-C (PQ-C)	0.0318	0.0466	***0.55***
Carotenoids	Echinenone	0.0094	0.0161	***0.78***
Carotenoids	α-tocopherol	0.0712	0.0423	−0.75
Carotenoids	Echinenone	24.6293	22.0992	−0.16
Carotenoids	Canthaxanthin (all trans)	3.2139	5.3849	***0.74***
Carotenoids	Canthaxanthin (9- or 13-cis)	1.8934	3.6241	***0.94***
Carotenoids	Phytoene (isoform 1)	0.2167	0.2115	−0.04
Carotenoids	Phytoene (isoform 2)	0.1211	0.1672	0.47
Carotenoids	Phytoene (isoform 3)	0.3874	0.3568	−0.12
Carotenoids	Lutein+Zeaxanthin	0.1903	0.1517	−0.33
Carotenoids	Didehydro-β-caroten-2-one (or other isoforms)	0.1185	0.0756	***−0.65***
Carotenoids	Didehydro-β-caroten-2-one (or other isoforms)	0.0619	0.0517	−0.26
Chlorophyll	Pheophorbide a	0.3058	0.0683	***−2.16***
Chlorophyll	Pheophytin	4.2824	2.4453	***−0.81***
Galactolipid	Monogalactosyl diacylglycerol (MGDG; 34:1)	6.2429	4.7038	−0.41
Galactolipid	Monogalactosyl diacylglycerol (MGDG; 32:2)	5.9388	3.131	***−0.92***
Galactolipid	Monogalactosyl diacylglycerol (MGDG; 34:3)	15.0015	12.1347	−0.31
Galactolipid	Monogalactosyl diacylglycerol (MGDG; 36:1)	0.0242	0.0419	***0.8***
Galactolipid	Monogalactosyl diacylglycerol (MGDG; 36:2)	2.3625	1.6715	−0.5
Neutral lipid	Triacylglycerol (TAG; 46:0)	0.0428	0.0131	***−1.71***
Neutral lipid	Triacylglycerol (TAG; 48:0)	0.0368	0.0098	***−1.91***
Neutral lipid	Triacylglycerol (TAG; 52:0)	0.0363	0.0102	***−1.83***
Neutral lipid	Diacylglycerol (DAG; 51:2)	0.7083	0.588	−0.27
Neutral lipid	Fatty acyl esters of hydroxy fatty acid (FAHFA; 52:1)	1.3111	1.0269	−0.35
Phospholipid	Phosphatidylserine (PS; 38:0)	0.0587	0.0328	***−0.84***

^a^ Lipid annotations are based on accurate mass matches and annotations/identifications made in Susanto, et al. 2025.

^b^ Statistically significant fold-changes are indicated with bold, italic text. Student’s t-test (p < 0.05), n = 4 independent biological replicates.

Principal Component Analysis (PCA) of the cyanoglobule lipidome revealed a clear separation between control and nitrogen-deficient samples (**Fig 3A**), indicating robust and reproducible differences in lipid composition. Broad class-level remodeling was observed (**Fig 3B**), with quinones becoming the dominant lipid group under nitrogen deficiency, comprising over 40% of the total annotated lipid pool. This shift was driven by significant enrichment of plastoquinone derivatives, particularly PQ-B; C16:0 (log2 FC 1.45), PQ-B; C18:1 (1.23–1.27), and PQH_2_-E; 18:2 (1.04). The accumulation of these redox-active lipids suggests an adaptive response to oxidative stress, consistent with the identification of Glutaredoxin and NDC1 (ndbB) in cyanoglobules [39–42].

Among the plastoquinones, PQH_2_-E; 16:0 and 18:1 were among the most abundant species under both conditions, while plastochromanol-8 (PCH_2_–8) − a reduced cyclized derivative of PQ-9 − showed a modest increase (log2 FC 0.16). Canonical PQ-9 (*i.e.*, PQ-A) also rose by ca. 50%. Several minor variants, including PQ-C, increased moderately, supporting the idea that cyanoglobules function as dynamic reservoirs for redox lipids during nitrogen stress [39].

In contrast, chlorophyll-related metabolites were strongly depleted. Pheophorbide a, a major catabolite of chlorophyll breakdown, decreased by over 4-fold (log2 FC –2.16), and pheophytin by nearly 2-fold (log2 FC –0.81). These trends support the hypothesis that cyanoglobules play a role in chlorophyll turnover and degradation during stress, potentially aiding detoxification or recycling processes. This pattern also aligns with the visual chlorosis observed under nitrogen deprivation [29]. Carotenoid content showed selective changes. Echinenone, the most abundant carotenoid of the cyanoglobules, exhibited divergent regulation, with one isomer modestly increasing (log2 FC 0.78) while another decreased (log2 FC –0.16). Notably, canthaxanthin (9- or 13-cis) showed a nearly 2-fold increase (log2 FC 0.94), whereas α-tocopherol declined significantly (log2 FC –0.75), possibly reflecting increased oxidative stress consumption. These patterns echo changes at the plastoglobules in land plants, where carotenoids contribute to both structure and photoprotection [43,44].

Galactolipids, especially monogalactosyl diacylglycerol (MGDG), were broadly depleted. MGDG; 32:2 and MGDG; 34:1 declined by log2 fold-changes of –0.92 and –0.41, respectively. This likely reflects membrane lipid turnover and thylakoid remodeling. Interestingly, MGDG; 36:1 increased (log2 FC 0.80), suggesting differential regulation of specific lipid species. In contrast to *Synechocystis*, where galactolipids increase to support cyanoglobule proliferation under phosphate stress, their depletion in *Anabaena* may indicate thylakoid breakdown or lipid mobilization under nitrogen limitation.

Neutral lipids such as triacylglycerols (TAGs) showed a consistent and substantial decrease. TAG; 46:0, TAG; 48:0, and TAG; 52:0 all declined by log2 FC values between –1.7 and –1.9. This contrasts with findings in *Synechocystis*, where TAGs accumulate in cyanoglobules under phosphate deprivation, suggesting that TAG regulation in cyanoglobules is stress-specific and/or species-dependent.

Taken together, these findings define the nitrogen-responsive lipid architecture of *Anabaena* cyanoglobules. The pronounced enrichment of plastoquinone derivatives and specific carotenoids, combined with a reduction in chlorophyll catabolites, TAGs, and thylakoid-derived lipids, supports the view that cyanoglobules serve as specialized compartments for redox buffering and lipid remodeling during nutrient stress [5]. These results reinforce the emerging model of cyanoglobules as dynamic organelle-like structures, analogous to land plant plastoglobules, capable of reorganizing their lipid and protein content in response to environmental cues, promoting stress adaptation of the organism.

## Discussion

Cyanoglobules have emerged as metabolically relevant compartments in cyanobacteria, yet their roles in stress physiology and lipid metabolism remain underexplored, particularly in filamentous strains [7,30]. In this study, we provide the first detailed characterization of cyanoglobules in a non-nitrogen-fixing strain of *Anabaena sp. PCC 7120* under nitrogen deprivation. These findings demonstrate that cyanoglobules undergo significant morphological and biochemical remodeling in response to nitrogen stress, reinforcing their functional analogy to plastoglobules in plant chloroplasts and cyanoglobules in *Synechocystis*. Importantly, by using a non-diazotrophic background, this work reveals cyanoglobule behavior in the absence of alternate factors such as heterocyst differentiation and nitrogenase expression, offering a clearer view of cyanoglobule function as a core stress response rather than a byproduct of diazotrophic specialization.

The substantial increase in cyanoglobule size and abundance of *Anabaena* under multiple stresses mirrors earlier observations in *Synechocystis* under phosphate stress [7], as well as plastoglobules in land plants under environmental stress [5,45,46]. These data support the idea that cyanoglobule formation is a conserved, nutrient stress-induced adaptation, independent of the presence or absence of heterocysts. While lipid droplets in *Anabaena* have historically been interpreted through the lens of nitrogen fixation [47–49], our results with a ΔN strain provides direct evidence that cyanoglobule formation occurs broadly within vegetative cells, suggesting a generalized role for cyanoglobules in cellular homeostasis during nitrogen limitation.

The proteomic analysis revealed that the cyanoglobule core proteome shares commonalities with that of land plant plastoglobules, encompassing redox regulators such as NDC1 (*alr4094/ndbB*), carotenoid-related enzymes such as β-carotene 4-monoketolase (*all3744/crtO*), and structural or regulatory components including PG18 (*alr4514*), FBNβ (*alr4318*), and ABC1K3 (*all4960/spkH*). The pronounced enrichment of these proteins under nitrogen deficiency suggests that cyanoglobules undergo substantial redox and lipid remodeling in response to nutrient stress. Importantly, these changes occurred even in the absence of heterocyst differentiation, indicating that cyanoglobule biogenesis is a direct response to nitrogen deprivation rather than a secondary consequence of diazotrophic development. Collectively, these findings position cyanoglobules as autonomous metabolic compartments that dynamically respond to environmental cues, reinforcing their evolutionary and functional continuity with plant plastoglobules [7,47].

Lipidomic profiling also supports this view. Cyanoglobules in the *Anabaena*^ΔN^ strain accumulated a suite of plastoquinone derivatives (PQ-B, PQH_2_-E), which are implicated in stress signaling and reactive oxygen species (ROS) buffering [50–53]. These compounds have also been identified in plastoglobules and cyanoglobules of *Synechocystis* [7], strengthening the argument for evolutionary and functional conservation. In contrast, the depletion of thylakoid-associated lipids (*e.g.*, MGDG, chlorophyll catabolites) and TAGs in response to N-deficiency suggest roles in lipid turnover and stress-driven remodeling rather than simple storage. Notably, TAGs decreased significantly in *Anabaena*^ΔN^ cyanoglobules during nitrogen deprivation − diverging from the accumulation pattern seen in phosphate-stressed *Synechocystis* − which may reflect distinct metabolic demands or regulatory networks across species or stress conditions.

By isolating and analyzing cyanoglobules in a non-nitrogen-fixing filamentous strain, we show that these structures can be studied without the overlay of heterocyst-specific metabolism, enabling cleaner attribution of molecular responses to the stress itself. This simplified framework makes the *Anabaena*^ΔN^ strain a useful experimental context for dissecting cyanoglobule function in filamentous cyanobacteria. Understanding cyanoglobule dynamics in this context may inform future efforts in engineering cyanobacteria for bioproduction or stress-resilient traits.

Finally, while the composition of cyanoglobules was characterized using proteomics and LC-MS/MS based lipidomics, there are important limitations to consider. Lipid quantification depends on ionization efficiency, which varies among lipid species and may result in the underrepresentation of low-abundant or highly polar lipids. Despite these technical constraints, the consistent identification of conserved plastoglobule homologs, redox-related enzymes, and stress-responsive proteins, along with reproducible lipid shifts across biological replicates, provides strong support for the biological relevance of the findings.

In summary, this work defines the cyanoglobules of *Anabaena* sp. PCC 7120 as dynamic and stress-responsive compartments that are morphologically and compositionally akin to plant plastoglobules and the cyanoglobules from another cyanobacterial model species, *Synechocystis* sp. PCC 6803 [7]. By focusing on a non-diazotrophic strain, we demonstrate that key features of cyanoglobule formation and remodeling are observed independently of heterocyst differentiation and nitrogenase activity. These findings help reveal how cyanoglobules may function as specialized compartments in cyanobacteria, similar to plastoglobules in plant chloroplasts. They also highlight how photosynthetic organisms, both prokaryotic and eukaryotic, use compartmentalized lipid structures to maintain redox balance and adapt to nutrient stress.

## Supporting information

S1 Fig Circular representation of genomic variants identified in *Anabaena* strains. (PDF)

S2 Fig Microscopic investigation of *Anabaena*^ΔN^ cyanoglobules under various stresses. (PDF)

S3 Fig Growth curve of *Anabaena*^ΔN^ showing changes in chlorophyll a content, cell density (OD₇₅₀), and cyanoglobule (CG) number per cell over time. (PDF)

S4 Fig Morphology of *Anabaena*^WT^ and *Anabaena*^*Δ*N^. (PDF)

S1 Table SNP analysis of *Anabaena*^WT^ and *Anabaena*^△N^ genome sequences. (XLSX)

S2 Table Cellular ultrastructure measurements of *Anabaena*^*Δ*N^. (XLSX)

S3 Table Proteomic analysis of isolated cyanoglobules. (XLSX)

S4 Table Lipidomic analysis of isolated cyanoglobules. (XLSX)

S1 File S1_raw_images. (PDF)
