# Supplementary material for: Cyanoglobule lipid droplet accumulation as a stress response to nitrogen starvation in a non-N2-fixing mutant strain of Anabaena sp. PCC 7120
Source: PLoS One. 2026 Feb 20;21(2):e0343220. doi: 10.1371/journal.pone.0343220 (PMC12923008; doi:10.1371/journal.pone.0343220)
Supplement: S1 Fig — (PDF) [file pone.0343220.s001.pdf]

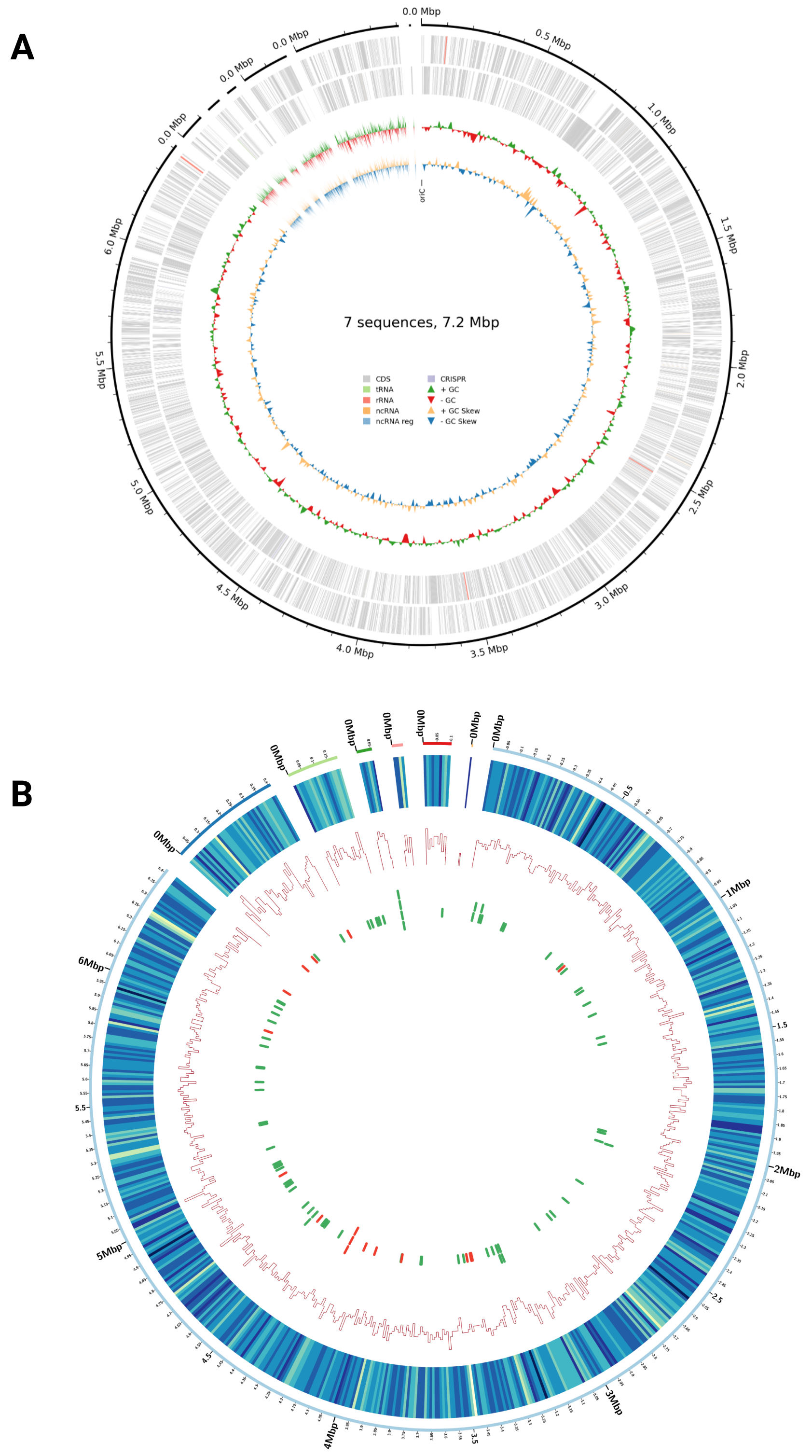

Supplementary Figure S1. Circular representation of genomic variants identified in *Anabaena* strains. Panel **A** corresponds to the wild type (*Anabaena*^{WT}) genome, and panel **B** shows the *Anabaena*$^{\Delta N}$ mutant strain. In panel B, the ring displays annotated genomic features, with the inner green marks indicating single nucleotide polymorphisms (SNPs) and small insertions/deletions (InDels) detected from whole-genome sequencing. The green lines specifically highlight mutation sites identified through SNP calling analysis, as detailed in Supplementary Table S1. The genome size corresponds to ~7.2 Mbp distributed across seven replicons.
