# Supplementary material for: Cyanoglobule lipid droplet accumulation as a stress response to nitrogen starvation in a non-N2-fixing mutant strain of Anabaena sp. PCC 7120
Source: PLoS One. 2026 Feb 20;21(2):e0343220. doi: 10.1371/journal.pone.0343220 (PMC12923008; doi:10.1371/journal.pone.0343220)
Supplement: S2 Fig — (PDF) [file pone.0343220.s002.pdf]

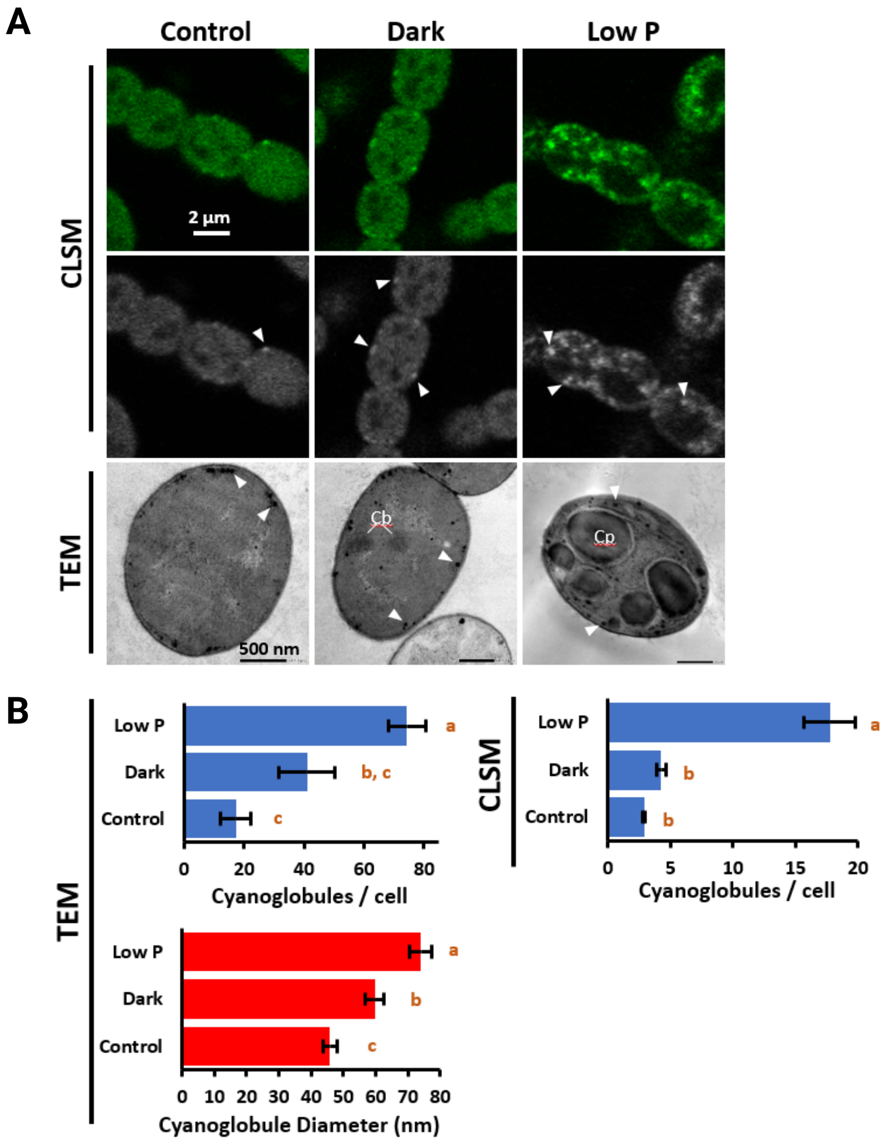

Supplementary Figure S2. Microscopic investigation of *Anabaena*$^{\Delta N}$ cyanoglobules under various stresses. (A) Representative confocal laser scanning microscopy (CLSM) and transmission electron micrographs (TEM) grown to stationary phase (control and P-deficient) or for seven days under darkness (dark treatment). Cells were stained with the fluorescent dye, monodansyl pentane, for CLSM observation. White arrowheads indicate representative cyanoglobules observed in CLSM and TEM. Cb, carboxysome. Cp, cyanophycin granule. Scale bars indicate 2 µm (CLSM all to the same scale) or 500 nm (TEM). (B) Quantification of cyanoglobule abundance (blue bar graphs) or cyanoglobule size (red bar graphs). Bars represent the mean ± 1 s.e.m. For measurement of cyanoglobule abundance , n = 3 biological replicates (independent cultures) each comprising the average value from at least 15 individual cells For measurement of cyanoglouble size, n = 15 cyanoglobules (cyanoglobule diameter). Letters indicate statistically significant differences by one-way ANOVA at 95% confidence using the Tukey multiple comparison test.
