# Supplementary material for: Cyanoglobule lipid droplet accumulation as a stress response to nitrogen starvation in a non-N2-fixing mutant strain of Anabaena sp. PCC 7120
Source: PLoS One. 2026 Feb 20;21(2):e0343220. doi: 10.1371/journal.pone.0343220 (PMC12923008; doi:10.1371/journal.pone.0343220)
Supplement: S3 Fig — (PDF) [file pone.0343220.s003.pdf]

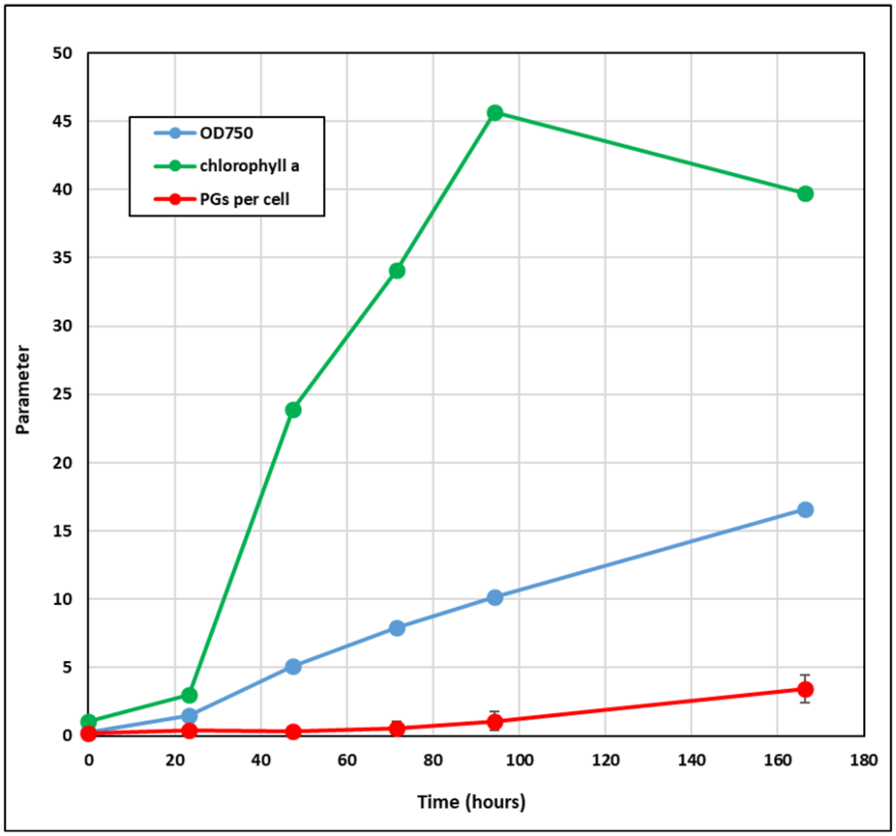

Supplementary Figure S3. Growth curve of *Anabaena*$^{\Delta N}$ showing changes in chlorophyll a content, cell density (OD_{750}), and cyanoglobule (CG) number per cell over time. Cultures were grown under $BG11^{-N}$ conditions, and samples were collected at the indicated time points for measurements. OD_{750} (blue) represents cell density, chlorophyll a (green; µg per mL) indicates photosynthetic pigment accumulation, and cyanoglobules per cell (red) reflect cyanoglobule proliferation during growth.
