# Supplementary material for: Cyanoglobule lipid droplet accumulation as a stress response to nitrogen starvation in a non-N2-fixing mutant strain of Anabaena sp. PCC 7120
Source: PLoS One. 2026 Feb 20;21(2):e0343220. doi: 10.1371/journal.pone.0343220 (PMC12923008; doi:10.1371/journal.pone.0343220)
Supplement: S4 Fig — (PDF) [file pone.0343220.s004.pdf]

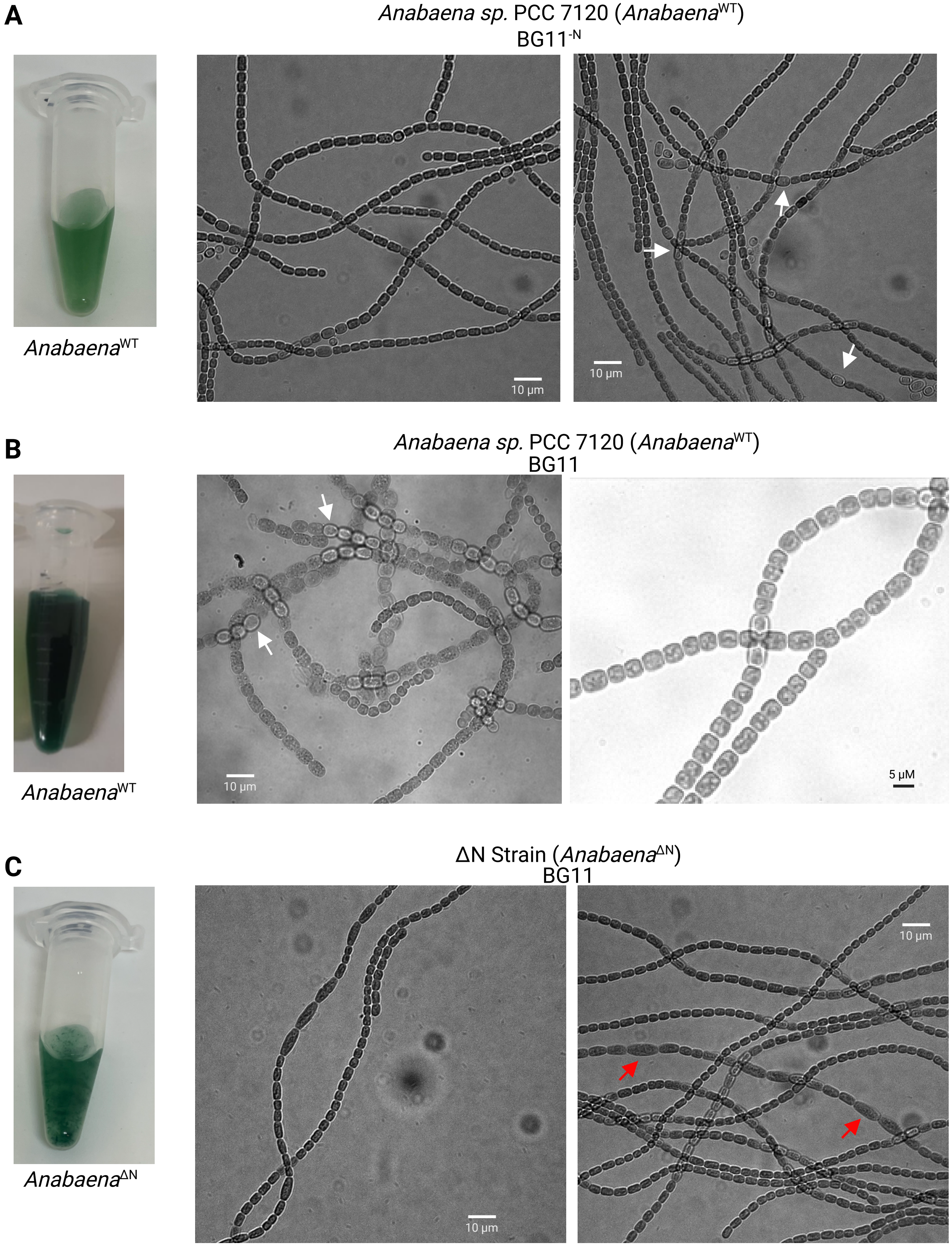

Supplementary Figure S4. Morphology of *Anabaena*^{WT} and *Anabaena*^{ΔN}. (A) *Anabaena*^{WT} grown in BG11^{-N} medium lacking nitrogen supplementation. Differentiated heterocysts are visible and indicated by white arrows. (B) *Anabaena*^{WT} grown in BG11 medium exhibiting typical vegetative filament morphology and formation of heterocysts (white arrows). (C) The *Anabaena*^{ΔN} strain, grown in normal BG11 media, exhibits variable morphology, including both normal-appearing cells and frequent elongated or swollen cells (red arrows). In liquid culture, the *Anabaena*^{ΔN} strain also displays a clumpy phenotype (see left image), in contrast to the homogeneous green suspension observed with *Anabaena*^{WT}.
