# Supplementary figures and images for: Cyanoglobule lipid droplet accumulation as a stress response to nitrogen starvation in a non-N2-fixing mutant strain of Anabaena sp. PCC 7120
Source: PLoS One. 2026 Feb 20;21(2):e0343220. doi: 10.1371/journal.pone.0343220 (PMC12923008; doi:10.1371/journal.pone.0343220)

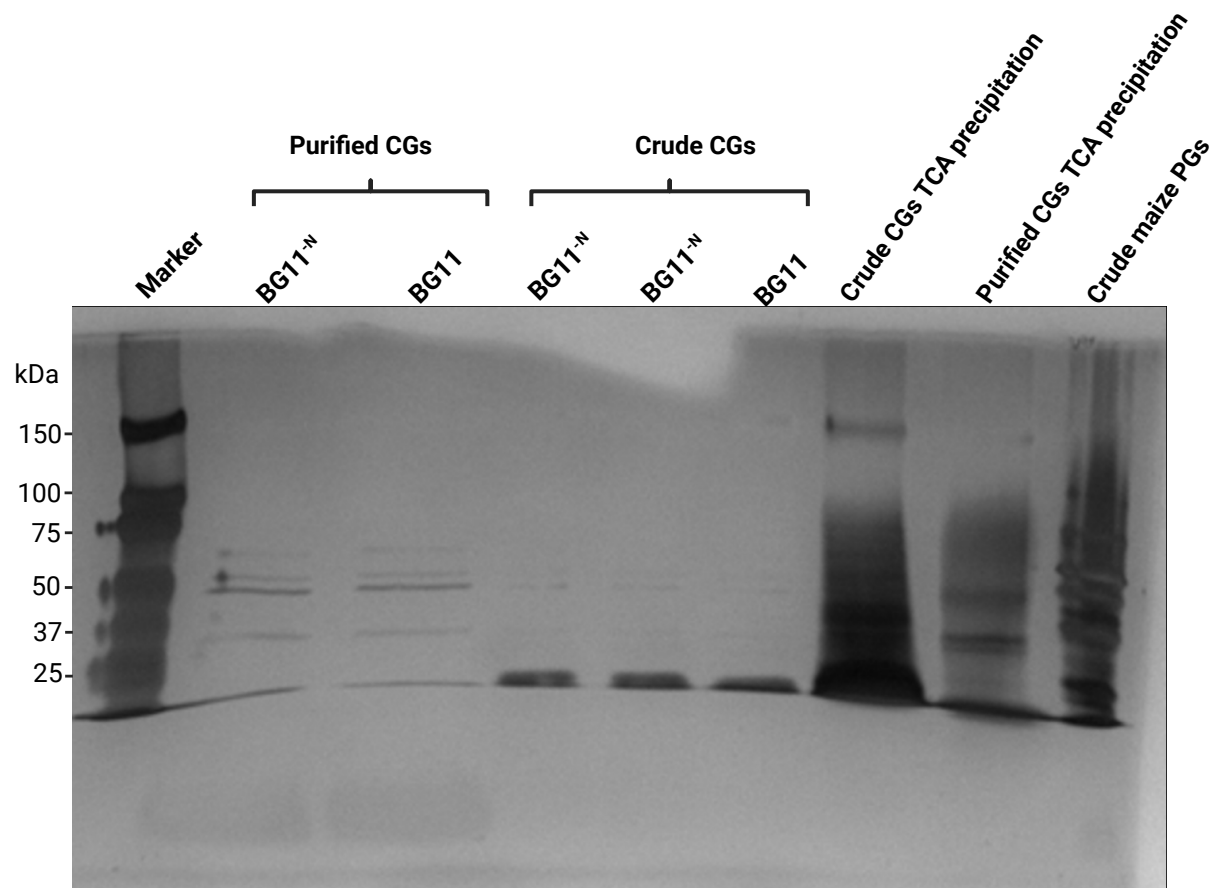

Supplement: S1 File — (PDF) [file pone.0343220.s009.pdf]
